# Perspectives on gastric cancer: the central role of gastrin in carcinogenesis by *Helicobacter pylori*, oxyntic atrophy and drug-induced acid suppression

**DOI:** 10.3389/fonc.2026.1807681

**Published:** 2026-07-28

**Authors:** H. Waldum, R. Fossmark, I. Modlin

**Affiliations:** 1 Department of Clinical and Molecular Medicine, Faculty of Medicine and Health Sciences, Norwegian University of Science and Technology, Trondheim, Norway; 2 Department of Gastroenterology, Oslo University Hospital, Oslo, Norway; 3 Department of Surgery, St. Olav’s Hospital-Trondheim University Hospital, Trondheim, Norway; 4 Yale University School of Medicine, New Haven, CT, United States

**Keywords:** acid, carcinogenesis, enterochromaffin like (ECL) cell, gastric cancer, gastrin, *Helicobacter pylori*, inflammation

## Abstract

**Background:**

The function of the acid production in the upper gastrointestinal tract is to kill swallowed microorganisms and has been preserved in development since primitive fishes. However, acid production contributes to peptic ulcers and reflux esophagitis.

**Main body:**

Gastric acidity is sensed by the G cell producing gastrin, which regulates release of mediators from and proliferation of the enterochromaffin like (ECL) cell. Drugs inhibiting gastric acidity profoundly induce hypergastrinemia and were early shown to cause malignant tumors in rodents. In humans these drugs also lead to hypergastrinemia and ECL cell hyperplasia, but tumors have not been reported until more recently. Tumor latency depends on the natural life length of a species. H. pylori, the main cause of gastritis, peptic ulcer disease and gastric cancer, was early accepted as a carcinogen by WHO. However, no direct carcinogen was found and subsequently H. pylori carcinogenesis was shown to depend on development of oxyntic atrophy which is not compatible with being a direct carcinogen. H. pylori and autoimmunity are the main causes of oxyntic atrophy, hypoacidity, hypergastrinemia and gastric cancer.

**Conclusions:**

Gastrin is central in gastric carcinogenesis. H. pylori is the dominating cause of gastric cancer of both intestinal and diffuse types and H. pylori carcinogenesis may be explained by hypergastrinemia. A gastrin antagonist is long-awaited.

## Introduction

Acid production in the upper gastrointestinal tract initially developed in primitive fishes and was preserved during evolution. Its role in food digestion is well recognized as well as its critical role in the eradication of swallowed microorganisms. The clinical significance of gastric acidity is indirectly reflected in its well described harmful effects that culminate in peptic ulcer disease and reflux esophagitis. The central role of gastric acid in the pathogenesis of these diseases is reflected in the efficacy of drugs causing inhibition of acid secretion.

The histamine-2 blocker cimetidine, introduced in the early seventies (1) represents the first efficient inhibitor of gastric acid secretion, and the first efficient drug in the treatment of peptic ulcer. Over the next decade and certainly by the mid-eighties far more effective inhibitors, including the irreversible H-2 blocker loxtidine (2), and the first proton pump inhibitor (PPI) omeprazole (3) had been described.

In 1985 long-term rodent studies reported that both these compounds induced gastric tumors localized to the oxyntic mucosa (4, 5). These lesions were initially classified as neuroendocrine tumors (NETs) originating from the enterochromaffin-like (ECL) cell driven by the hypergastrinemia (6) as a consequence of pharmacologically induced hypoacidity. The company Glaxo discontinued further development of loxtidine while Astra persisted with the production of omeprazole. The decision made by Astra was based upon the acceptance by physicians that the tumors observed in rodent stomachs were of little significance for humans. This assertion was widely accepted although viewed controversial by some. A notable expert in the field of acid induced disease, Kenneth Wormsley summed up the divisiveness of issue in his pithy remark: “This is the first compound giving cancer in its target organ in animals, accepted for clinical use”. Over the subsequent four decades the issue of the risk of profound acid inhibition with respect to gastric cancer has remained controversial and of concern.

This current evaluation seeks to address the topic of the pathological consequences of profound and sustained acid suppression with secondary hypergastrinemia and the biology of gastric tumors. The present review considers gastric physiology in humans and animals, gastric pathology, clinical gastroenterology and gastric endocrinology. The lifelong sustained tumor derived hypergastrinemia of individuals with gastrinoma is also examined to provide insight into the conundrum of gastrin driven neoplasia. Finaly, the role gastrin in H. pylori gastric carcinogenesis, the dominating cause of the disease, is also discussed.

## Gastrin and acid secretion

Gastrin is a regulator of acid secretion via the antral – ECL axis. Gastric acidity is sensed by the G cell producing gastrin and the antral hormone initiates release of histamine from the ECL cell, which finally stimulates the secretion of hydrochloric acid (HCl) from the parietal cell. Gastrin was early recognized as a trophic regulator of the oxyntic mucosa based largely on the description of gastrinoma by Zollinger and Ellison in 1955 (7) and their description illustrated the close relationship between the functional and growth-promoting effects of hormones.

To further understand the mechanisms by which gastrin might promote carcinogenesis, attention turned to identifying the cell types that express the gastrin receptor. By the early 1970s, histamine had been established as a regulator of gastric acid secretion (1) and Håkanson et al. demonstrated that the ECL cell produced this histamine (8). Concurrently with these developments, considerable effort was devoted to investigating the interactions among the three principal stimulators of gastric acid secretion: acetylcholine, gastrin, and histamine. In 1976, Berglindh and colleagues reported the isolation of rabbit oxyntic glands and oxyntic mucosal cells, enabling the assessment of parietal cell acid secretion through measurements of radioactive 5-aminopyrine accumulation and indirectly by oxygen consumption (9). It was noted that a cholinergic agent and histamine in contrast to gastrin stimulated acid secretion (9) indicating that gastrin did not have a direct stimulatory effect. Moreover, Rangachari using oxyntic mucosa in a Ussing chamber similarly found that gastrin stimulated acid secretion via inducing histamine release (10). Similarly, in the totally isolated rat stomach model gastrin, in contrast to a cholinergic agent, did not stimulate maximal histamine stimulated acid secretion (11) whereas gastrin was an efficient stimulator of histamine release in the same model (12). At this point it was evident that from a functional point of view the gastrin receptor was not localized to the parietal cell, but to the ECL cell which produced histamine. This concept was further supported by clinical observations.

## Classification of gastric cancers

Classifications of tumors should reflect cancer biology and have a clinical relevance. Determination of the cell of origin of cancers is therefore of both theoretical and clinical value.

Anatomically the stomach mucosa may be divided into three types: cardiac, oxyntic and antral, based upon the localization and differences in glandular structure and cellular types. The greater part of the gastric mucosa comprises the acid producing oxyntic mucosa and is characterized by oxyntic glands containing the parietal cells, chief cells and the neuroendocrine regulatory ECL cells. There is a gradual caudal transformation of the cardiac mucosa to the oxyntic mucosa, and oxyntic glands may even be identifiable deep in the antral transition zone (13). Typically, the antral glands lack parietal and chief cells, and the main neuroendocrine regulatory cell is the gastrin producing G-cell (13).

### Morphological classification

Laurén classified gastric adenocarcinomas into two categories; intestinal(expressing glandular structures) and diffuse lacking such structure (14). The diffuse type was broadly included among adenocarcinomas based upon its positivity for periodic acid-Schiff (PAS) which was erroneously believed to be mucin specific (15). However, a re-assessment of PAS specificity by immunohistochemistry demonstrated that PAS positive material in gastric cancer cells was negative for mucin but positive for neuroendocrine markers (16). Although there are other classification systems of gastric carcinomas, the classification of Laurén may reflect significant biological differences as the two cancer types do not transform into each other (14). Furthermore, the decline in incidence of gastric carcinomas noted during recent decades is exclusively due to a reduced frequency of the intestinal carcinoma type (17). Other morphological classification systems of gastric carcinomas like the WHO classification have not added significantly to the understanding of carcinogenesis and is also of questionable value in guiding for treatment (18), therefore not discussed in this review.

### Molecular classification

Gastric cancers have also been classified by two molecular platforms, the Asian Cancer Research Group (ACRG) and The Cancer Genome Atlas Consortium (TCGA). Both classification systems were compared with the Lauren system, but without defining a new class of microsatellite instability (MSI) which is associated with excellent effects of immunotherapy and favorable prognosis, the clinical impact of the classification has otherwise been marginal (19).

## Cell of origin

Two main theories have been proposed regarding the cell of origin: transformation of a proliferating stem cell (20) or dedifferentiation of a mature cell that acquires increased proliferative capacity. Genetic changes may be induced by external factors such as radiation or genotoxic agents, but increased cell proliferation per se also increases the risk of malignancy as each cell division carries a small risk of mutation (21). Sustained proliferative stimulation therefore increases both the likelihood of acquiring mutations and the expansion of mutated clones.

The observation that ECL cells frequently occur in small clusters has early been considered suggestive of self-replication. The question of whether ECL cells could divide was solved in studies of rats by Tielemans and Willems as well as by Håkanson with co-workers in the 1990s, by combining histamine immunocytochemical staining with autoradiography after *in vivo* labelling with (3H) thymidine (22, 23). They demonstrated that ECL cells proliferated under gastrin control. Sheng et al. found that immature, but not mature mouse ECL cell could divide based upon Ki67 expression (24), and Hayakawa et al. described that Mist1 cells could give rise to immature ECL cells (25). Apparent discrepancies concerning the proliferative capacity for mature ECL cells may be attributed to their low rate of proliferation which may fall below the sensitivity of the methods employed. Since both mature and immature ECL cells are capable of proliferation, and gastrin plays a central role in regulating this process, these observations support a role for ECL cells in gastric carcinogenesis.

### The ECL cell as putative origin of diffuse type gastric cancer

**Figure 1:**
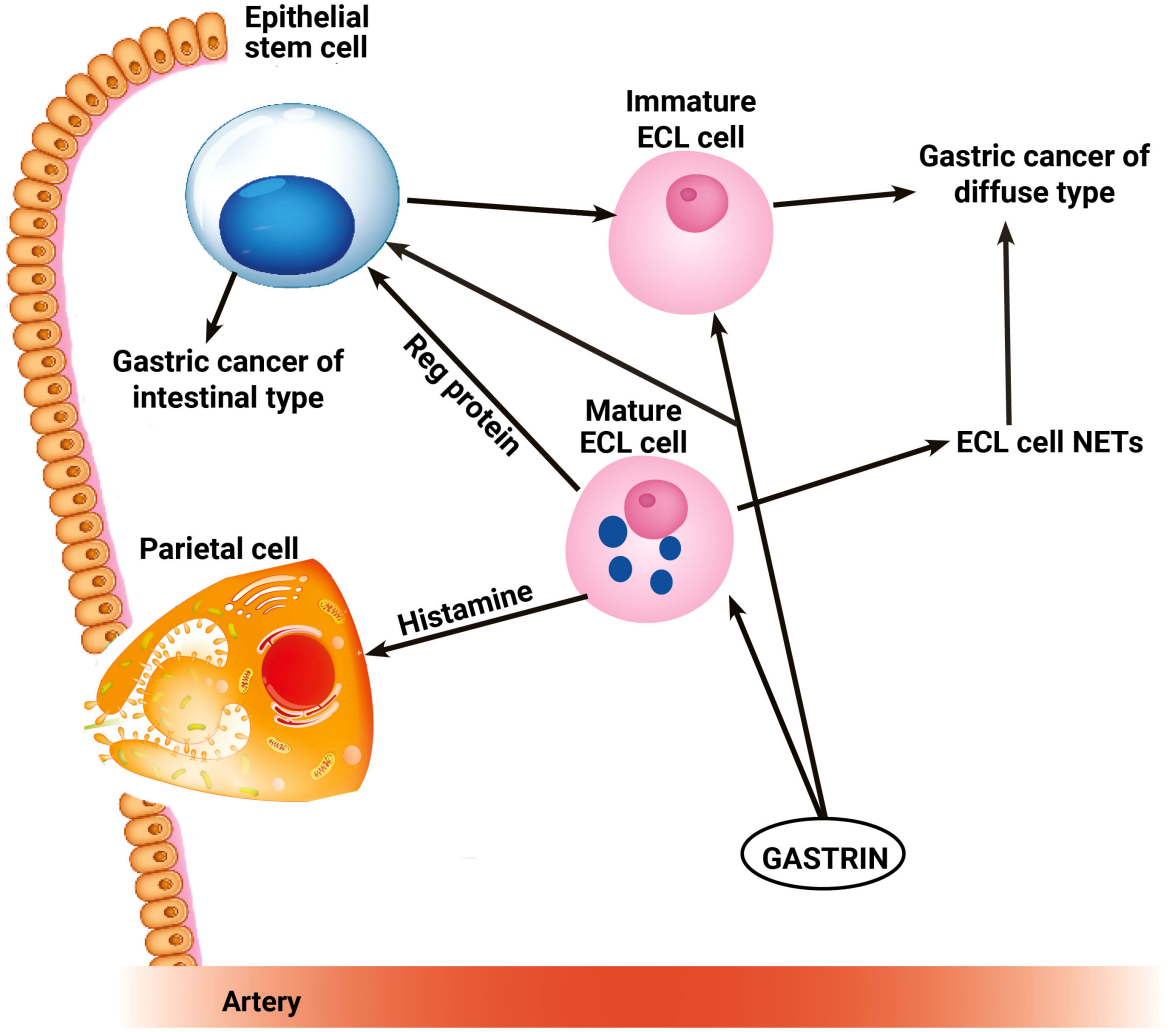
Putative role of gastrin and the enterochromaffin-like (ECL) cell in the regulation of gastric acid secretion and in gastric carcinogenesis (reproduced by permission from Waldum HL, Sørdal ØF, Mjønes PG. The Enterochromaffin-like [ECL] Cell—Central in Gastric Physiology and Pathology. Int J Mol Sci. 2019;20 (10):2444. Doi: 10.3390/ijms20102444 (26)). Modified by introducing immature ECL cells according to Sheng W, Malagola E, Nienhüser H, Zhang Z, Kim W, Zamechek L, et al. Hypergastrinemia Expands Gastric ECL Cells Through CCK2R(+) Progenitor Cells via ERK Activation. Cell Mol Gastroenterol Hepatol. 2020;10 (2):434-49.e1 (27).

Although the epithelial stem cell is often considered as the cell of origin of adenocarcinomas, the observations that intestinal and diffuse types of gastric cancer do not interconvert (14) as well as epidemiological differences may indicate otherwise. Such discrepancies may be explained by diffuse and intestinal gastric carcinomas arising from different cells of origin. A common mediator could promote carcinogenesis through direct stimulation of one cell type while simultaneously inducing the release of a substances that facilitates malignant transformation of another cell type. (**Figure 1**) (26). The illustrated model has support from numerous studies suggesting that ECL cells may develop into tumors of moderate degree of malignancy both in animals and humans (4, 5, 27–31). In an early study of expression of NE markers in human gastric cancer cells, NE markers were found in 40% of diffuse type gastric carcinomas, in some tumors in virtually all carcinoma cells, but in none of intestinal type carcinomas (32). Although the report was considered controversial at first (33), the specificity of the methods used was confirmed (34). In a larger follow-up study, surgical specimens from consecutive patients with gastric cancer were examined by immunohistochemistry, immune electron-microscopy and hormonal concentrations and at least 10% of the gastric carcinoma cells expressed ECL cell markers (35). These findings were confirmed in subsequent studies (36), using immuno-histochemistry (32), immune-electron microscopy and *in-situ* hybridization, and carcinoma cells were found to express NE and ECL cell markers, secretory granules (35, 37) and gastrin receptors (38), respectively. Furthermore, signet ring cell carcinomas examined by immunohistochemistry and *in-situ* hybridization, and the amorphous material in the cytoplasm was found to be negative for mucin, but positive for NE markers (39), further supporting that these tumors should be classified as NE. Despite the evidence that ECL cells have the capacity to proliferate, it was nevertheless proposed that human ECL cells are incapable of proliferation and, consequently, unable to give rise to malignant tumors (40). This assertion has also been contradicted by the description of a gradual histological progression from ECL cells, through successive stages of ECL-cell NET development, into a highly malignant diffuse-type gastric carcinoma accompanied by gradual loss of NE markers, in a patient with pernicious anemia (41). Since immature (25) or mature (41) ECL cells appear to have a central role in gastric carcinogenesis (42), the driver hormone gastrin seems likely of having a central role in gastric carcinogenesis.

However, Hayakawa et al. (43) have reported that the oxyntic epithelial stem cell expressed Mist1 and probably being the cell of origin both for diffuse and intestinal types of gastric cancers. Although Mist1 cell may represent the epithelial stem cell population in the oxyntic mucosa (43), the gradual transition of gastric NET to carcinoma (41) as well as the expression of neuroendocrine/ECL cell markers in gastric carcinomas in patients with hypergastrinemia (44) indicate that ECL cells nevertheless are important in gastric carcinogenesis. It seems to be a discrepancy between the findings based on modern biological methods on mice cells and traditional immunohistochemical examination of human gastric tumors. In 2017 Hayakawa, Fox and Wang (45) reviewed the origin of gastric cancer based on their own work and while they focused on stem cells (46) they accepted that cancers could arise from some long-lived differentiated cells. In their review, the authors noted that, in mice, gastrin promotes the development of gastric cancer in the corpus (45). Furthermore, the development of gastric cancer in the context of *H. pylori* infection, has been attributed to an early event in the course of infection, although the underlying mechanism remains unknown (45). Although, there are several years since the Hayakawa paper (45), we cannot see that there has been any new breakthrough in this field.

### CCK2R biology

The CCK2R is central in gastric physiology, and it is well-known that the CCK2R is expressed both in the oxyntic and antral mucosa. In the oxyntic mucosa CCK2R is expressed mainly on the ECL cell where gastrin via CCK2R regulates the acid secretion by release of mediators like histamine, and proliferation by Reg proteins (47). The presence of a gastrin receptor on the parietal cell has been disputed since the sixties and seemed to be clarified by its cloning from gastric mucosal cells fortified in parietal cells (48). However, gastrin has no effect on maximal histamine-stimulated acid secretion (11) and has a marked and higher proliferative effect on the ECL cell than on other oxyntic mucosal cells including the parietal cell (49). Hormones may influence the growth and tumorigenic potential of neighboring cells indirectly through the release of secondary mediators that act on cells expressing the corresponding receptors, even when those cells do not express the original hormone receptor. The difference in trophic effect on the ECL cell compared with the other oxyntic mucosal cells (49) may be explained by an indirect gastrin stimulation of epithelial stem cell proliferation via ECL cell mediators or by a direct effect via a gastrin receptor on the stem cell. Thus, from a functional point of view there is no need for a gastrin receptor on the parietal cell. Gastrin stimulates the release of histamine from the ECL cell, which stimulates parietal cell secretion of acid, but since the general trophic effect on oxyntic cells is preserved with histamine depleted ECL cells, histamine seems to have no general proliferative effect on the gastric mucosa (50). Moreover, the Mist1 cell does not express the CCK2/gastrin receptor (43). Already in 2001 Kazumori et al. described expression of the CCK2 receptor on proliferating cells in the neck zone of gastric fundic glands and that these cells were not parietal, ECL or D cells (51). Hypergastrinemia induces clusters of ECL cells apparently developing from mature ECL cell, but mainly by stimulating the proliferation of younger ECL cells developing from epithelial isthmus stem cell (24). The fact that gastrin has a general trophic effect on the oxyntic mucosal cells and a specific and more marked effect on the ECL cell (49) implies that gastrin stimulates the proliferation of a cell giving rise to a cell being able to differentiate into all these cells.

In the antrum gastrin receptors have been described on stem cells, which interacts with progastrin and claimed to be involved in carcinogenesis (52). The regulation of the antral stem cell via progastrin may require future reconsideration since prohormones seldom have biological function. A biological role of progastrin has been suggested for long and was revied rather recently (53). However, no hard data indicating a real biological effect is still missing which is to be expected taking into consideration how complicated regulation of biological functions by hormonal fragments would be. Because oxyntic glands are also found deep into the antrum (13) gastrin receptors transmitting effects like those in corpus are also expected to be present in parts of the antrum. Finally, rather than grouping cells together based solely on the expression of a single receptor (e.g., CCK2R-positive cells), it is preferable to identify and refer to each cell type individually among those that express the receptor.

### Single- cell and spatial transcriptomic studies

Single cell and spatial evidence have rather recently been applied in studying gastric cancer (54). By this method Tsubosaka et al. identified a new stem cell marker in the gastric mucosa, LEFTY1 (55). The expression and localization of the different types and subtypes in oxyntic mucosa were identified including neuroendocrine cell subtypes, but unfortunately without ECL cell markers (55). Thus, this study cannot provide information of the ECL cell as cell of origin of gastric cancer. However, isthmus stem cells can differentiate into all lineages of gastric mucosal cells including the ECL cell (43) and immature ECL cells expressing the CCK2R are stimulated to proliferation by hypergastrinemia (24) possibly predisposing to neoplasia. Thus, the ECL cell whether mature or immature, may nevertheless be central in gastric carcinogenesis. Moreover, Mist1 expression is a marker of human chief cells and was reported to be lost in metaplasia, dysplasia and carcinoma (56). Finally, the identification of receptors regulating proliferation on putative stem cells would be important.

## The role of gastrin in human gastric carcinogenesis

At the time of the description in rodents of gastrin dependent ECL cell derived tumors secondary to long-term profound acid inhibition (4, 5), it was also realized that a similar mechanism had been described in humans (28). The reports of gastrin induced ECL cell NETs in patients with autoimmune gastritis were regarded as “benign” and of little clinical relevance. In 1973 McGuigan and Trudeau described increased gastrin levels in the blood of individuals with gastric cancer, except those having a prepyloric cancer who exhibited reduced gastrin (57). Decades later, in a prospective study gastrin was elevated in persons who later developed gastric cancer (58).

The two most common causes of gastric cancer, *H. pylori* infection and autoimmune gastritis, share a similar pathogenic pathway consisting of inflammation of the oxyntic mucosa with atrophy, reduced acid secretion and secondary hypergastrinemia. The two conditions are discussed below.

### The putative role of gastrin in *H. pylori* carcinogenesis

**Figure 2:**
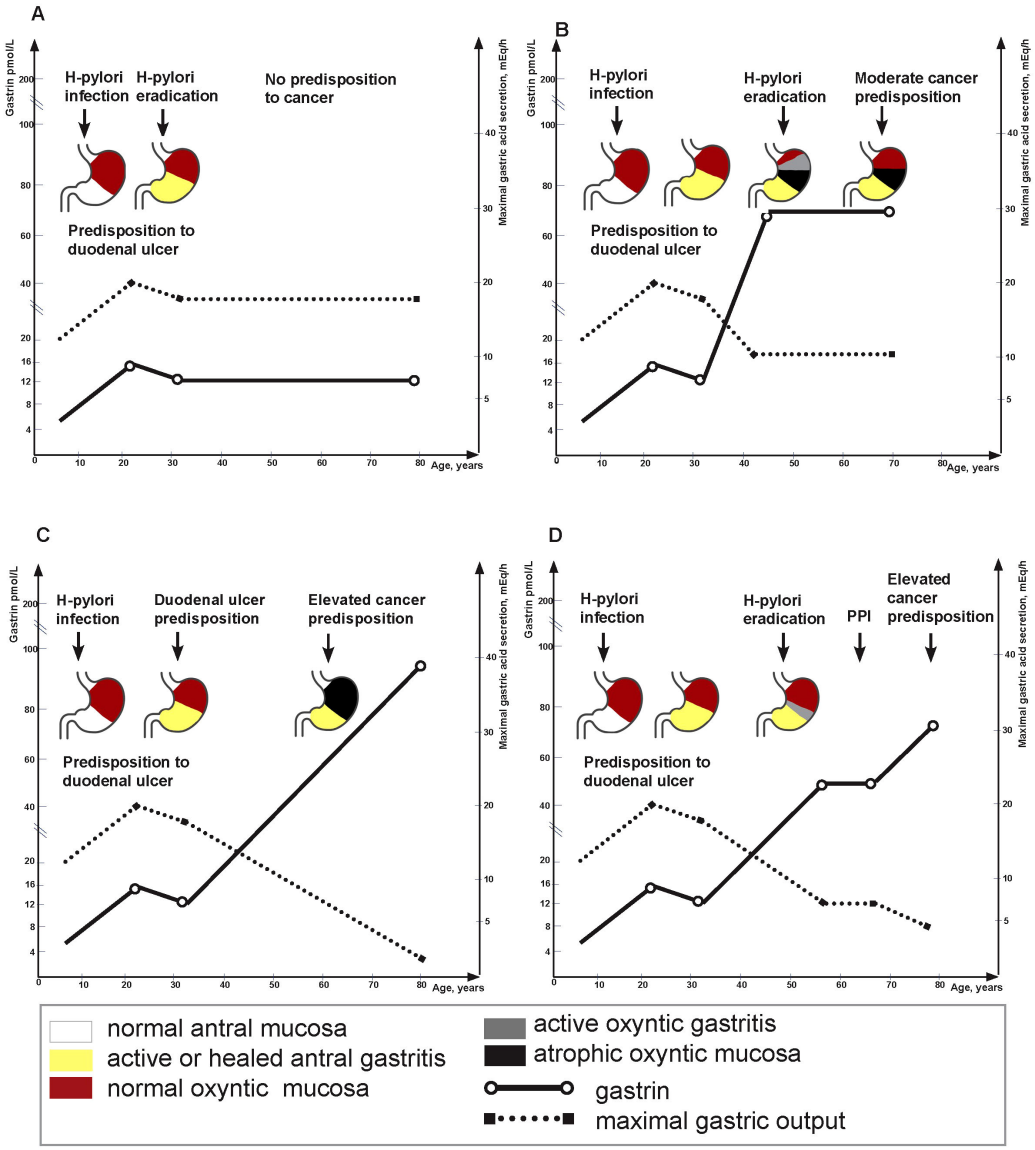
The consequences of Helicobacter pylori gastritis which initially affects the antral mucosa causing a stimulation of gastrin release resulting in a minimal increase in gastrin which due to its potency is sufficient to stimulate acid secretion and thus predispose to duodenal ulcer **(A)**. The inflammation creeps orally successively affecting more and more of the oxyntic mucosa **(B)** thereby and by induction of atrophy result in progressive reduction in acid secretion leading to gastric hypoacidity and hypergastrinemia **(C)**. By the trophic effects of gastrin on the enterochromaffin like (ECL) cell and indirectly on the other mucosal cells of the oxyntic mucosa, long-term hypergastrinemia predisposes to gastric carcinomas of diffuse as well as intestinal types. Profound inhibitors of gastric acid secretion in subjects with Helicobacter pylori oxyntic gastritis or in patients with oxyntic atrophy where Helicobacter pylori have been eradicated will have an increased risk of gastric cancer **(D)**. Reproduced with permission from Scand J Gastroenterol. 2025;60:652-663. Doi: 10.1080/00365521.2025.2509094 (Ref (42)).

Since Marshall and Warren’s initial reports it has become apparent that H. pylori is a predominant cause of gastric cancer and that oxyntic atrophy was a prerequisite for H. pylori gastric carcinogenesis (59). WHO early recognized H. pylori as a carcinogen as most experts considered it as a direct carcinogen. Nevertheless, despite intensive research, no such carcinogen has been found although H. pylori with *cytotoxin associated gene* (Cag)A appears more carcinogenic than H. pylori without this gene (60). Both Cag A positive and negative H. pylori infection predispose to gastric cancer (61). However, in Mongolian gerbils Cag A positivity of H. pylori was very important for inducing gastritis (62), thus it may be that the higher incidence of gastric cancer in persons with Cag A positive H. pylori strains reflects a more severe gastritis. The hypothesis that H. pylori is a direct gastric carcinogen should thus have been reconsidered when evidence was presented that H. pylori carcinogenesis was dependent of oxyntic atrophy (59). Further observations have also been presented that suggest the initial hypothesis is incompatible viz: H. pylori cause duodenal ulcer, a condition without major hypergastrinemia and reduced risk of gastric cancer (63); individuals in whom H. pylori infection has been eradicated are predisposed to gastric cancer of diffuse type decades after the bacterium was removed (64, 65) and exhibit a higher cancer risk when associated with extensive gastric atrophy (66). H. pylori eradication at a stage of only mild atrophy compared with severe atrophy is more efficient in reducing the gastric cancer risk (67), and the preventive effect of previous duodenal ulcer on H. pylori carcinogenesis seems to be preserved also for cancers after H. pylori eradication (68). However, the increased incidence of gastric carcinoma with a relative short latency in patients treated with drugs inducing profound acid inhibition after H. pylori eradication (69), suggests an additive effect via hypergastrinemia. In 2009 gastrin was accepted as an essential cofactor for H. pylori associated gastric corpus carcinogenesis in C57BL/6 mice (70), and in 2015 we hypothesized that gastrin could mediate the carcinogenic effect of H. pylori infection (71). In conclusion, it is evident that H. pylori does not have a direct carcinogenic effect whereas many of the current aspects of H. pylori gastric carcinogenesis may rather be mediated by gastrin (42). H. pylori oxyntic gastritis predisposes to both cancer of diffuse and intestinal types (72) in agreement with the view that gastrin stimulates the ECL cell to proliferate as well as release mediators stimulating other cell proliferation. Reg protein released from ECL cells is the most plausible candidate as an indirect stimulator of cells in the oxyntic mucosa (47) and has been shown to stimulate proliferation (73). **Figure 2** illustrates the interaction of gastrin in H. pylori gastric carcinogenesis. In summary, *H. pylori* initiates gastric carcinogenesis by inducing oxyntic gastritis and atrophy, leading to reduced acid secretion and secondary hypergastrinemia. The resulting chronic stimulation of cell proliferation increases the likelihood of accumulating mutations, ultimately promoting the development of gastric neoplasia with varying degrees of malignancy.

The role of H. pylori in carcinogenesis of the cardia is more variable with differences between people from Asia and Europe (74). Interestingly, oxyntic glands and ECL cells are inconsistently expressed in the cardia (75), which may support the weaker association between H. pylori and cardia cancer compares to in the oxyntic mucosa (76). An important argument against a central role of gastrin in gastric carcinogenesis is the belief that gastric cancers occurs more distally in people from Asia compared with Europeans. Studies confirming this is, however, not yet available, but H. pylori gastritis has been reported to be predominately localized to the antrum in people from Korea and Japan (77). However, in Japanese patients ECL cells were reported to be present in the intermediate and pyloric regions (78). Whether there are quantitative differences between people from East and West with respect to ECL cell distribution with possible consequences for carcinogenesis remain to be determined. As gastric NETs regress after 12 weeks treatment with the gastrin antagonist netazepide (79) and given the knowledge from animal studies, this antagonist could be useful in early stages of H pylori carcinogenesis in patients. However, since a gastrin antagonist is still not available, the final role of gastrin in H. pylori carcinogenesis cannot be proven. Furthermore, it must be stressed that a role for gastrin in gastric carcinogenesis depends on the presence of oxyntic mucosa with ECL cells expressing the gastrin receptor. As stated above, the variable role of H. pylori in cardia carcinogenesis may be explained the scarcity of oxyntic glands. Similarly, it may appear contradictory that gastric cancer occurs as often in the antrum as the corpus. However, oxyntic glands are most often also present, even deep in the antrum (13). It has been suggested that antral inflammation for instance by H. pylori could lead to increased occurrence of ECL cells in the antral mucosa (so-called antralization of oxyntic glands), but any publication describing this is difficult to find. Nevertheless, the study by Choi et el from the Goldenring group (13) demonstrates that there are sufficient ECL cells in the antral mucosa to play a role in gastric carcinogenesis.

### Autoimmune gastritis

Autoimmune gastritis is an inflammation of the oxyntic mucosa which finally leads to mucosal atrophy with secondary hypergastrinemia due to loss of acid secretion. It has been suggested that gastric cancer in autoimmune gastritis could reflect an early and transient H. pylori infection (80). This is contradicted by the observation of reduced risk of gastric cancer in patients with duodenal ulcer which is caused by H. pylori but nevertheless protects against cancer (63, 81). Indeed, a recent review concluded that autoimmune gastritis demonstrates typical features of autoimmune disease and is unrelated to H. pylori (82). The risk of gastric NETs as well as carcinomas is increased in individuals with autoimmune gastritis (83–85), with a relative risk of gastric cancer seven times higher compared with the background in an Italian population (86). The reported cumulative incidence of gastric NETs and gastric cancers was 4% and 3%, respectively, after seven years observation time in a Finnish cohort (85). Interestingly, by using a highly sensitive immunohistochemical method (tyramide signal amplification) most of the gastric cancer cells in patients with autoimmune gastritis expressed NE and ECL cell markers (44).

Although the pivotal role of gastrin in the development of ECL cell–derived gastric NETs is widely recognized, the development of gastric carcinomas has traditionally been explained primarily by the direct carcinogenic effects of chronic inflammation. Several mechanisms may in theory account for the association between persistent inflammation and carcinogenesis (87). (1) The cause of the inflammation is carcinogenic itself. H. pylori carcinogenesis depends on oxyntic atrophy and is accordingly not a direct carcinogen. Concerning autoimmune gastritis, the cause of the proposed autoimmunity is unknown. (2) Substances released from inflammatory cells may be genotoxic. This has not been convincingly shown. (3) The cell death induced by the inflammation induces compensatory proliferation and increases the risk of mutations. (4) Changes in cell composition due to inflammation may modify functions, resulting in hormonal changes causing selective alterations in proliferation. Loss of parietal cells results in gastric hypoacidity and secondary hypergastrinemia leading to specific ECL cell hyperstimulation. In a recent comprehensive review, Massironi et al. discussed potential tumorigenic factors in autoimmune gastritis, including genetic and epigenetic alterations, inflammatory mediators, microbial changes, and their interaction with hypergastrinemia secondary to parietal cell loss (88). However, the initiating event in autoimmune gastritis remains unknown. Although antibodies against parietal cells are useful diagnostic markers, they are not considered pathogenic, and parietal cell loss is believed to result from cellular immune mechanisms. Importantly, there is no clear evidence that parietal cells are affected before other oxyntic mucosal cells. Indeed, a study of early, pre-atrophic autoimmune gastritis demonstrated inflammation in the lamina propria without parietal cell destruction or atrophy (89). Thus, the parietal cell may not be the primary target but rather particularly vulnerable to inflammatory injury because of its high metabolic demands and specialized acid-secretory function. Despite numerous hypotheses, the underlying cause of autoimmune gastritis remains unresolved.

Although gastric NETs occur both together with H. pylori gastritis (90, 91) as well as autoimmune gastritis (29, 92), gastric NETs are far more common in patients with autoimmune gastritis (93). In autoimmune gastritis, patients with the highest gastrin levels have the greatest risk of developing NETs (94). The higher prevalence of NETs in autoimmune gastritis compared with H. pylori-induced atrophic gastritis may therefore reflect more pronounced hypergastrinemia. This is likely related to the more extensive oxyntic atrophy typically seen in autoimmune gastritis (95).

### Oxyntic atrophy as common pathway

Gastric oxyntic atrophy implies a definitive and important risk factor for gastric cancer in both autoimmune gastritis (96) and H. pylori gastritis (97). H. pylori are the most prevalent cause of oxyntic atrophy, and although H. pylori infection is more common in Eastern than in Western countries, the difference in gastric cancer prevalence is higher than being explained by prevalence of H. pylori infection alone (98). A particular strain of Cag A+ H. pylori in Eastern-Asia causes severe gastritis and seems to be especially important in carcinogenesis (98), while in other parts of Asia may have a high prevalence of H. pylori infection yet a low prevalence of gastric cancer (99). Palli focused on the imperfect association between the prevalences of gastric cancer and H. pylori infection and the lack of detection of any carcinogen in H. pylori and advised research focusing on atrophic gastritis (100). In H. pylori infected children at mean age of 10.1 years gastric atrophy was evident in 6.4% (101). It is known that H. pylori eradication reduces the cancer risk as well as atrophy in long-term Intestinal metaplasia is closely linked to gastric atrophy and has been regarded as a higher-risk stage in the Correa cascade of gastric carcinogenesis (102). However, because intestinal metaplasia does not develop in the absence of atrophy, its independent carcinogenic role remains debated, and it may instead reflect long-standing atrophic changes (103). Gastric oxyntic atrophy may slowly regress when H. pylori is eradicated (104), which may be the main beneficial effect of eradication, as oxyntic atrophy was early linked to cancer risk after H. pylori eradication (105).

### Biomarkers of oxyntic atrophy and ECL cell hyperplasia

Oxyntic atrophy is largely asymptomatic. Given its cancer risk, a simple and reliable test to identify patients who should undergo upper endoscopy with biopsies for diagnosis and risk stratification would be useful, but no ideal marker exists. Pepsinogen I (PG I), produced by chief cells in the oxyntic mucosa and measurable in serum, is typically low in complete oxyntic atrophy (106). However, in active gastritis, inflammation-driven leakage of intracellular contents may result in normal or elevated levels, potentially masking partial atrophy (107). The combination of PG I with pepsinogen II (PG II) is also theoretically limited, as *H. pylori*-related atrophy may involve both antrum and corpus. Recent data suggest that serum PG I may help distinguish autoimmune from *H. pylori*-associated gastritis (108). Gastrin in blood regulates and reflects gastric acidity and is highly elevated in persons with anacidity (109). Moreover, after food intake gastrin increases to levels that are dependent on the meal constituents as well as capacity of the oxyntic mucosa to produce acid. In general, 24-h gastrin measurement is a better method to assess gastrin exposure than fasting gastrin though it is clinically impractical as a biomarker (110). When dosing a PPI once daily, the fasting morning gastrin may be normal (111). Gastrin is very potent resulting in a significant stimulation of histamine release and acid secretion at a concentration of 2 pmol/L in the isolated rat stomach (112, 113). Moreover, the trophic effect of gastrin is primarily on the ECL cell with a steep concentration response curve reaching near maximal stimulation at a concentration of about 100 pmol/L in rats (114). A similar concentration response curve of gastrin assessed indirectly by acid secretion (115) and ECL cell density as found in humans (29). Accordingly, maximal effective trophic gastrin concentration is in the range of 200–300 pmol/L. Regarding gastrin measurement, the specificity of the antibodies used in the immunoassay is important, and previously established “normal” ranges are likely set too high, as they have included individuals with H. pylori gastritis (116).

The ECL density is regulated by gastrin exposure during 24-h. Therefore, an ECL cell mass marker would indirectly reflect gastrin activity. Histamine is unsuitable due to rapid degradation, while chromogranin A, though not ECL-specific, is elevated in ECL cell hyperplasia/neoplasia (117, 118). Its utility of chromogranin A during PPI therapy may improve if baseline levels are obtained before treatment initiation.

## ECL cell NETs, ECL cell cancers and adenocarcinomas

In 1971 Soga et al. described an argentaffin cell adenocarcinoma of the stomach which initially had been classified as a well-differentiated adenocarcinoma (119) demonstrating the difficulty of classification without using more specific staining of markers. A few years earlier Soga had reclassified the gastric tumors in Mastomys from adenocarcinoma to gastric NETs (120). A similar reclassification from gastric carcinoma to gastric neuroendocrine neoplasia (gastric NEN) was later in the same decennium reported for 10 of 140 gastric cancer tumors (121). A direct transformation of a gastric carcinoid to an adenocarcinoma has also been observed (122). Moreover, it is well-known that mixed tumors showing both endocrine and non-endocrine differentiation exist (123, 124). In the same line is the endocrine differentiation in signet ring cell carcinomas (16) as well as in gastric carcinomas of patients with pernicious anemia (44). Thus, it should be clear that to reach a precise diagnosis and differentiation between endocrine and non-endocrine derived gastric cancers specific staining should be included. Furthermore, NETs may progress to highly malignant carcinomas (41, 125),In a recent international study the clinical profile of autoimmune gastritis differed between countries and regions as well as prevalences in gastric NETs and gastric cancer which depended on the level of gastrin and previous PPI use (126). In patients with autoimmune gastritis particularly those with high gastrin develop gastric NETs (94). Important is also the study reporting that patients with gastric NETs have an increased risk of gastric cancer (127).

## Hypergastrinemia and gastric neoplasia in animals

*Rats.* Rats given the insurmountable histamine 2-receptor antagonist (H2RA) loxtidine were in 1985 reported to develop profound hypergastrinemia and ECL cell carcinoids after life-long administration (5). Similarly, the proton pump inhibitor omeprazole was reported to induce a 15-fold increase in plasma gastrin (128), increase in ECL cell density and oxyntic mucosal thickness after ten weeks administration (129). Omeprazole also led to ECL cell hyperplasia (22) and carcinoids after life-long administration (4) and it was hypothesized that profound gastric hypoacidity and secondary hypergastrinemia was the mechanism behind the observed tumors. Subsequent studies documented the ability of rat ECL-cells to self-replicate in response to gastrin (130). Several other methods of inducing hypergastrinemia in rats were also explored, this included partial corpectomy (131, 132) and administration of the competitive H2-blocker ranitidine in high doses (133) which both led to carcinoid formation. Ciprofibrate, a peroxisome proliferator-activated receptor α agonist, was also found to induce ECL cell carcinoids (134) in rats through a direct effect on the antral G-cell (135) and without concomitant gastric hypoacidity (136). These studies further strengthened the argument that hypergastrinemia per se is carcinogenic, not caused by one particular drug or limited to the hypo-acidic state.

*Mice.* The effects of hypergastrinemia in mice has been studied after administration of acid inhibiting drugs as well as in genetically modified mice. Loxtidine was in 1986 found to induce ECL cell carcinoids after two years administration (137), identical to the reported findings in rats (5). Rather recently an increase in gastric ECL cells secondary to hypergastrinemia in mice was confirmed (24). However, long-term administration of omeprazole did not result in development of any tumors (4), but omeprazole was administered to mice in the same dose as in rats (400 *μ*mg/kg/day) under the assumption that the dose was sufficient to affect gastric acidity and gastrin concentration. It has later been documented that mice require much higher doses of omeprazole (1750 *μ*mg/kg/day subcutaneously) to induce profound hypoacidity and hypergastrinemia (138) and long-term effects in mice have so far not been investigated with appropriate study design. However, there is a synergistic interaction between hypergastrinemia and H. pylori infection in a mouse model of gastric cancer (139).

Various genetically modified mice models have been generated. Transgenic INS-GAS mice overexpress gastrin and resulting in a four-fold increase in plasma gastrin and hypersecretion of gastric acid, thus modelling human gastrinomas (139). While young INS-GAS mice have increased ECL cell numbers at 3 months age, older INS-GAS mice lose parietal cells and identifiable ECL cells and a proportion develop tumors with adenocarcinoma phenotype in the oxyntic mucosa. Inoculation with *Helicobacter felis (H. felis)* further elevates plasma gastrin and accelerates carcinogenesis considerably (139). The problem with the INS-GAS model is that it is difficult to differentiate the role of the initial moderate hypergastrinemia due to the gene manipulation and the secondary oxyntic atrophy.

Subsequent studies of the carcinogenetic mechanism found that loxtidine as well as the gastrin receptor antagonist YF476 (later named netazepide) (140) inhibited tumor formation.

A comprehensive study of INS-GAS mice, gastrin deficient mice and C57BL/6 wild-type mice infected with *H. felis*, aiming to isolate the effects of gastrin in *H. felis*-induced carcinogenesis concluded that gastrin was essential in the pathogenesis of gastric corpus cancer (70). E.g. *H. felis* did not induce dysplasia in gastrin deficient mice, while carcinogenesis was accelerated in INS-GAS mice.

The gastric proton pump consists of two subunits and the effects of knocking out each of them have also been studied. H+K+ATPase β subunit-deficient mice have a 4- to 7-fold increase in gastrin secondary to gastric anacidity, accompanied by marked oxyntic mucosal hypertrophy (141, 142). H+K+ATPase β subunit and gastrin double knockout mice have gastric anacidity but do not develop hypertrophic oxyntic mucosa (141), demonstrating that gastrin is responsible for the observed changes in H+K+ATPase β subunit deficient mice. Although H+K+ATPase β subunit deficient mice have ECL cell hyperplasia, carcinomas in the oxyntic mucosa are rare and without expression of NE markers (143). As expected, H+K+ATPase α subunit deficient mice develop changes very similar to β subunit-deficient mice (144).

Furthermore, knockout of the gene encoding the potassium voltage-gated channel regulatory subunit 2 (KCNE2) also results in achlorhydria-induced hypergastrinemia and pronounced hyperplasia of the oxyntic mucosa in mice 3 months of age (145). Adenomas with dysplasia have been described after 16 weeks (146), whereas longer-term studies have not been published. In patients with the rare Jervell and Lange-Nilsen syndrome due to potassium channel dysfunction, hypergastrinemia has been observed in most patients and in a subgroup of five patients who underwent upper endoscopy, gastric mucosal hyperplasia/dysplasia was found in three of the patients (147).

The enzyme histidine decarboxylase (HDC) synthesizes histamine and gastric changes in HDC-deficient mice have been studied. They are hypo-acidic with a threefold elevation of plasma gastrin (148), gastric anacidity and marked increase in corpus mucosal thickness and NE cell density after nine months (149). Histamine-2 receptor deficient mice are also hypergastrinemic, but have a hypertrophic oxyntic mucosa with an increase in NE cells (150). Long-term studies addressing the carcinogenic effects of hypergastrinemia in the absence of histamine have not been published, however, these studies suggest that histamine mediates a general trophic effect of gastrin, but not via the histamine-2 receptor.

Biological gastrin effects may be removed by ablation of the gastrin cells which can be done with diphtheria toxin or by surgical removal of the main source of gastrin, the gastric antrum. Moreover, the gastrin receptor may also be ablated and thus removing any biological gastrin effect. Gastrin cell ablation results in apparently normal mice, with the only change in the stomach with reduced number of ECL cells and parietal cells. The lack of effect on other oxyntic mucosal cell types than the ECL cell and the parietal cell by gastrin cell ablation does not indicate a gastrin receptor on the presumptive Mist1 epithelial stem cell. Moreover, acid secretion was disproportionally reduced taking the ECL and parietal cell numbers into consideration indicating unresponsiveness (151) (152, 153).

Experimental ablation of gastrin-producing cells or gastrin receptors has provided additional insight into the mechanisms underlying gastric carcinogenesis. Studies involving targeted deletion of the gastrin gene or its receptor in mice have demonstrated altered gastric physiology and mucosal architecture, underscoring the importance of gastrin signaling in maintaining normal cellular homeostasis. These models have highlighted that loss of gastrin activity can lead to reduced ECL cell proliferation and may impact the development of neoplastic lesions, further supporting the central role of the gastrin-ECL cell axis in gastric pathology.

*Mastomys natalensis.* The African rodent *Mastomys natalensis* was already in the 1950s described to develop multicentric gastric tumors (154, 155). The tumors in the particular Mastomys strain were first classified as adenocarcinomas but were in 1969 reclassified as neuroendocrine tumors (120, 156) and later more specifically identified as ECL-cell tumors (157, 158). Multicentric tumors developed in up to 50% of animals 1–2 years of age and were located in the gastric corpus. The histological changes preceding the tumor development were seen in three stages: 1) linear hyperplasia of ECL cells, 2) ECL-cell nodules restricted to gastric glands and 3) ECL- cell growth below the lamina propria (159). The exact mechanism behind carcinoid development in Mastomys is uncertain, but it has also been found that Mastomys have a CCK2/gastrin receptor with enhanced basal level of activity compared to the human gastrin receptor (160). However, we have not found any description of the gastric pH in Mastomys. Circulating gastrin is still considered essential for tumor development, since hypergastrinemia induced by loxtidine further promoted tumor formation as well as ECL cell hyperplasia in the surrounding mucosa (161). Furthermore, tumor formation can be prevented by a CCK2/gastrin receptor antagonist (YF476/netazepide) (162). Similar to the findings in Japanese cotton rats (below), the somatostatin-analogue octreotide has inhibitory effects on both gastrin cells and ECL cells and prevented loxtidine-induced ECL hyperplasia in Mastomys (163).

*Japanese Cotton Rats.* A strain of Japanese cotton rats was described to develop gastric carcinomas in the oxyntic mucosa in mainly female animals (164). Further studies revealed animals with tumors to have gastric hypoacidity, hypergastrinemia, ECL cell hyperplasia and tumors with ECL-cells using a range of markers and methods (30, 165, 166). Longitudinal studies found that a proportion of female cotton rats develop gastric hypoacidity of unknown etiology between age two and six months, hypergastrinemia and then carcinomas after approximately four months of hypergastrinemia. The tumors were prevented by administration of a gastrin receptor antagonist (YF476/netazepide), by a somatostatin analogue octreotide (167) or by antrectomy (168). In male cotton rats which do very rarely develop spontaneous tumors, ECL-cell hyperplasia and tumors could be induced by loxtidine (169) or by partial corpectomy (170), two different methods that induced hypoacidity and marked hypergastrinemia. Studies examining ultrastructural characteristics and NE markers in cell populations of the oxyntic mucosa revealed evidence of progressive dedifferentiation, characterized by the loss of NE granules and a gradual disappearance of various NE markers (171). This well-characterized rodent model of carcinogenesis in the gastric corpus illustrates that gastric hypoacidity and secondary hypergastrinemia may induce tumors with adenocarcinoma phenotype and NE characteristics.

*Mongolian Gerbils.* Mongolian gerbils or so-called desert rats inoculated with *H. pylori* have been used to study gastric carcinogenesis first reported in 1998 (172, 173). A combination of gastritis, ulcers, atrophy, intestinal metaplasia, and gastric carcinoma has been reported, mirroring the spectrum of changes caused by *H. pylori* infection in patients. *H. pylori* inoculation of gerbils results in a 5- to 10-fold rise in serum gastrin, which increases with time after infection (174), however, not all *H pylori* strains induce dysplasia and cancer to the same extent (175). Interestingly, Mongolian gerbils develop tumors with either adenocarcinoma or ECL cell carcinoid phenotype (172, 174, 176), which suggests common mechanisms and risk factors for two morphologically different types of neoplasia. Parallelling findings in Japanese cotton rats and INS-GAS mice, there is an increase in CgA positive cells up to week 50, which decreases from week 50 to 100 (177). Mucosal changes in Mongolian gerbils were gastrin dependent as illustrated by the preventive effect of gastrin receptor blockade (YF476/netazepide) (178). The effect of PPI in addition to H pylori infection has been explored in two different studies. Omeprazole and lansoprazole both increase gastrin levels if given in sufficient doses and PPIs increased the proportion of animals developing NETs (179) as well as adenocarcinomas (in 60% compared to 7% in controls animals after 6 months) (180).

*Dogs.* The Norwegian Lunde hund is a Norwegian spitz breed developed for hunting puffins (*Fratercula arctica*). Due to a canine distemper virus epidemic, the breed was nearly extinct in the 1940s, and the present population therefore originates from only five dogs. A significant proportion of Lunde hund suffer from the “Lunde hund syndrome” consisting of diarrhea, hypoproteinemia, ascites, subcutaneous oedema, weight loss, and lethargy (181). These Lunde hund also develop chronic atrophic gastritis and gastric tumors (182, 183), which are rarely seen in most dog breeds. Chronic atrophic gastritis is accompanied by parietal cell loss and linear hyperplasia of ECL cells (184). While carcinomas in dogs predominantly arise in the pyloric area, tumors in the Lunde hund most often develop in the corpus/fundus. Half the tumors have neoplastic cells with NE and ECL-cell differentiation (184). Thus, the combined findings parallel the development of gastric cancer in patients with autoimmune atrophic gastritis/pernicious anemia. The effect of long-term administration of an acid inhibitor has not been studied in the Norwegian Lunde hund.

Beagle dogs have been given omeprazole daily for 7 years (185) and the study found no changes in the gastric mucosa including ECL cell numbers. However, the dogs given omeprazole did neither have elevated fasting nor meal-stimulated plasma gastrin levels as compared to controls, meaning that they were not given a dose sufficient to test the effects of profound long-term acid inhibition.

## Hypergastrinemia secondary to surgery and inhibitors of gastric acid secretion

Several types of surgical procedures may lead to hypergastrinemia.

Like the procedure in rodents, partial resection of the corpus, while retaining the antrum may lead to hypergastrinemia and the effect of gastric sleeve resection as a bariatric procedure has been studied.

Gastric sleeve resections were initially found not to affect fasting or meal-induced gastrin release much compared to controls (186), and it could be hypothesized that since a proportion of the antrum was also resected and that intragastric pH was not significantly elevated. Subsequent prospective studies have documented significant within-patient increase in plasma gastrin 1 and 10 days after surgery as compared to baseline concentrations and a doubling of ECL cell numbers 6.2 years after surgery in five patients (187). Longer-term and larger human studies of gastric sleeve resection on ECL-cell density in patients have to the best of our knowledge not been studied.

Resection of the small intestine was reported to cause acid hypersecretion more than 100 years ago (188) and a tendency to develop peptic ulcers was early noted. It was later found that it was primarily the upper part of the small intestine that regulated acid secretion (189) and that hypersecretion also occurred after intestinal bypass (190). The mechanism involves loss of inhibitory feedback on acid secretion by gastric inhibitory polypeptide (GIP) and vasoactive intestinal polypeptide (VIP) (191), the latter by stimulating somatostatin release (192). Subsequent studies which included also pentagastrin stimulated acid secretion did not find increase secretion a median 5.3 years post-resection (193) and it has become evident that intestinal adaptation during the months following resection parallels the resolution of gastric acid hypersecretion. Long-term studies of gastric changes in patients having undergone jejunal resections are therefore less relevant.

## Hypergastrinemia secondary to inhibitors of acid secretion has been extensively studied

*The H2RA.* Both ranitidine and cimetidine were found to cause a slight and non-significant increase in fasting gastrin levels after eight weeks treatment (194). Tolerance to H2RA occurs after few weeks treatment (195) and the phenomenon includes attenuation of the effect on gastric acidity without further increasing plasma gastrin after day one. Long-term H2RA studies that have investigated ECL-cell density are missing, but patients using H2RA have been included as a comparative group in studies primarily focusing on the association between PPIs and gastric cancer. In a meta-analysis of five such studies, H2RA use was associated with gastric cancer (RR 1.32 (95% CI 1.08-1.62)) (196). However, in two separate studies, long-term H2RA treatment was not associated with gastric cancer (197, 198).

Proton Pump Inhibitors. PPIs are efficient inhibitors of gastric acid secretion and tolerance does not occur. The effect on intragastric pH varies between the different PPIs and depends on dose(mg) and dose interval (hours) (199, 200). Long-term use of PPI was evaluated in the LOTUS trial (201)) which noted that fasting gastrin levels five years after treatment start with a doubling of the median concentration. It was noted that a proportion of patients exhibited concentrations > 200 pmol/L. Other studies have found that hypergastrinemia in PPI users is more pronounced in females (202–204) and in individuals with mucosal atrophy (205). The reason for higher gastrin in females than males due to drugs causing profound acid inhibition in similar doses is not known but could just reflect the lower weight of women than males. Already in the late eighties PPI treatment was reported to induce ECL cell hyperplasia secondary to increased gastrin (206) similar to the initial changers seen in rats dosed with PPIs.

Mucosal changes have been reviewed and there is a consistent increase in ECL-cell linear and micronodular hyperplasia (207), including in the LOTUS trial with randomized design (208). The risk of developing corpus atrophy during PPI treatment was markedly higher in H pylori positive patients receiving treatment for reflux esophagitis (209)). This has been confirmed in a combination study (207). In a study of 20 people treated with conventional doses of PPIs for at least three years, variable hypergastrinemia was evident but only ECL cell hyperplasia and no ECL NETs or ECL carcinomas were identified (210). This may reflect the short observation time.

In contrast to the LOTUS study which reported two gastric polyps in 180 PPI users after five years (208), a Japanese prospective study of PPI users noted that 13.6% and 8.9% developed new fundic gland and hyperplastic polyps respectively (211).

Gastric neuroendocrine neoplasms *(gastric NENs)*. A series of case reports and smaller case series of patients with gastric NENs have been published (212, 213). A causal relationship between PPI use and gastric NEN development in these patients has been deemed probable by documentation of hypergastrinemia and by exclusion of autoimmune oxyntic gastritis and gastrinoma with gastric NET types 1 and 2 (189).

In a large population-based case control study in five Nordic countries with 1790 cases of gastric NENs and 17987 controls, PPI use was noted to be associated with gastric NEN in a dose-dependent manner, in particular when adjusted for various confounders such as atrophic gastritis or H pylori infection (214). Likewise, a marked temporal increase in gastric carcinoids during introduction of PPIs has been reported whereas the gastric cancer incidence was slightly reduced in the period 1981 to 2000 (215). Of particular relevance is the report that, family members with an inactivating mutation in the gene encoding the *H+K+ATPase* α-subunit were all noted to develop multiple gastric NETs in early adulthood with pronounced hypergastrinemia (31, 216). In one such individual a tumor with adenocarcinoma phenotype also developed, suggesting common pathogenetic factors in humans as well as in the previously discussed- animal models. This genetic condition resembles long-term PPI use and supports the view that long-term gastric hypoacidity resulting in hypergastrinemia induces gastric neoplasia. Very recently one paper described that long-term PPI use (> 5years) resulted in gastric NETs which they proposed should be classified as type 4 and that these tumors should have a good prognosis (217). Another similar study reported PPI induced gastric NETs with good prognosis after more than 1 year of drug use (218). It therefore seems overwhelmingly that long-term PPI use results in gastric NETs. Since gastric NETs like other tumors show the ability to become more malignant with time, the good prognosis seems doubtful.

Adenocarcinomas. The association between PPI exposure and a subsequent diagnosis of gastric cancer has been examined in numerous observational studies, case-control studies and cohort studies which have recently been extensively reviewed (219). An overview of 21 meta-analyses found that 20 identified a statistically significant increased risk of gastric cancer in PPI users. The largest effect was found in the most recent meta-analysis (RR = 2.88 (2.29-3.61) (220). One meta-analysis failed to identify a significant difference, but included only three clinical trials (OR = 1.06 (0.79-1.43)) (221). The meta-analyses have initiated controversy concerning the robustness of the published original studies and the presence of confounding factors. Issues raised include reverse causality and the choice of an appropriate lag time relevant to gastric carcinogenesis. While the potential effects of PPI use in individuals with healthy gastric mucosa may take decades to become evident, the associated risks may emerge more quickly in populations with preexisting lesions. In population-based studies, H. pylori status is unknown, and the H. pylori interaction cannot be assessed. Of special relevance therefore is the study where patients were treated with PPI prophylactically due to percutaneous coronary intervention since in this group there were few if any confounding factors. It is noteworthy that this study reported an increase in gastric cancer and death from gastric cancer in PPI users (222). Furthermore, a separate landmark study by Cheung et al. reported the risk of gastric cancer after H pylori eradication increased with duration of PPI use from HR 5.04 for ≥1 year to 8.34 for ≥3 years (69). Importantly, in this study, the use of H2RA was not associated with increased risk (HR 0.72, 95% CI 0.48 to 1.07).

The effect of PPI use in high-risk populations is illustrated by studies of patients after endoscopic treatment for early gastric cancer. In a large cohort study of 1,836 PPI users and 12,218 controls, PPI use was associated with metachronous cancer with a HR 5.5 95% CI 5.12-5.92) (223). Another study identified hypergastrinemia in PPI users, but not PPI use *per se*, to be a risk factor for metachronous PPI treatment to cancer (224). Gastric ECL cell hyperplasia and NETs were also linked to PPI treatment in another paper focusing on mucosal pathology (225). Very recently a Nordic study reported no increase in gastric adenocarcinomas in patients treated more than one year with PPI when adjusting for numerous covariates including previous H. pylori eradication treatment, peptic ulcer disease, smoking and alcohol related disorders (226). The minimum observation time seems to have been about five years but maximally 26 years and the PPI use the five years before inclusion was considered. Moreover, they did not specify the doses used in the various analyses. Efficient correction for H pylori infection, the main risk factor for gastric cancer, was not possible as opposed to in the study by Cheung et al (69). Taking into consideration the long latency for gastric cancer as shown by H. pylori, the exposure time of 1 year and observation time seem to have been too short.

In the epidemiological studies on the risk of malignancy due to long-term PPI use there has been a continuous problem with the role of reverse causation and confounding by indication (227). The authors have tried to minimize these problems by not including patients with relatively new symptoms, but in most cases uncertainty has persisted. In the present survey we have therefore based our argumentation on studies without problems with reverse causation or confounding by indication (214, 217, 218, 222).

Potassium competitive acid blockers (P-CABs) have been in clinical use in Japan from 2015 and were approved in the USA in 2023. In Europe, this class of acid inhibitory drugs has not yet been approved. The effect of a single dose is rapid and has a more pronounced effect on 24-h intragastric pH with time with pH ≥ 4 of approximately 90% (228). In fact, P-CABs properly dosed may induce virtually anacidity and thus potentially be used without antibiotics in the eradication of H. pylori (229). Whereas there is little doubt about the long-term efficacy, there is more concern about potential consequences of even more pronounced acid suppression. Treatment with vonoprazan 10 mg for 1–4 years caused a 7-12-fold increase in fasting gastrin, with severe gastric atrophy as a risk factor for higher concentrations (230). Analogous to the reports of conventional PPI use in females, females treated with vonoprazan exhibited higher gastrin concentrations (230). The reason for different degree of hypergastrinemia between sexes is not known but may just reflect similar drug doses although women have lower weight. In a comparative study, patients with mild or no atrophic gastritis who were treated with vonoprazan exhibited higher gastrin levels than those receiving conventional PPIs (231). In a five-year randomized controlled trial of erosive esophagitis treated with vonoprazan 10 mg, individuals exhibited three times higher fasting gastrin compared to those receiving lansoprazole 15 mg (625 pg/ml vs 200 pg/ml).We are not aware of any study where long-term PPI and P-CAB has been compared with respect to ECL cell NETs. The observation that the proportions of those with ECL cell hyperplasia did not differ (232) which may reflect the fact that the steep part of gastrin concentration/efficacy-curve had been reached at 200 pg/ml (114). Since the concentration-response curve for the trophic effect of gastrin on the ECL cell is rather steep and reaching near maximal effect at only moderate hypergastrinemia (16), the increased cancer risk of P-CABs compared with PPIs may not be dramatic. Hitherto, there is no report that P-CABs alone induce gastro NETs, but there exists one case report where PPI treatment for 11 years followed by P-CAB for 3 years induced gastric NETs (233). It has, however, to be taken into consideration that gastric NETs due to hypergastrinemia generally require decades to manifest themselves as shown for PPIs. Based on the differences in efficacy it is reason to believe that P-CABs will have a slightly higher risk of developing gastric NETs. On the other hand, the risk of gastric NETs due to acid inhibitory drugs also depends on the dose used.

## Gastrinomas and risk of secondary gastric neoplasia

ECL cell neoplasia have been classified as gastric NETs due to hypergastrinemia in patients with atrophic oxyntic gastritis (type 1) and due to gastrinoma (type 2) whereas NETs type 3 and NE carcinoma have been claimed to be gastrin independent (234). However, it is a misunderstanding that a tumor is independent of gastrin when developing during normogastrinemia (235). Gastrin can play a trophic/carcinogenic role even with near normal values as in Mastomys (161), and it should be noted that there is no threshold for the trophic effect of gastrin (236).

There are two types of gastrinomas, one related to a mutation in the MEN 1 gene coding for a tumor suppressor protein, Menin, predominantly effects neuroendocrine tissue, and the second type which is sporadic gastrinomas and without known aetiology (237). Individuals with gastrinoma as part of MEN 1 have higher risk of gastric NET compared with those with sporadic gastrinomas (238). It has been speculated that the genetic defect in the MEN 1 gene may exert a separate tumorigenic effect on the ECL cell not related to its effect on gastrin. Contrary to this viewpoint is the report that removal of all macroscopic gastrinomas in a patient with MEN 1 mutation resulted in disappearance of all ECL cell NETs (239). Nevertheless, the fact that gastrinomas of sporadic type predispose to gastric ECL cell NETs (238, 240, 241) strongly suggests that gastrin alone is sufficient to induce ECL cell tumors. Gastrinomas that are a component of MEN 1 also develop earlier in life and have a longer survival, which overall results in a longer duration of hypergastrinemia (240) compared with those with sporadic gastrinomas among which many may be cured by operation or die early due to their gastrinoma. This may partially explain the higher frequency of ECL cell NETs in the MEN I group.

## Implications for treatment of acid related diseases

**Figure 3:**
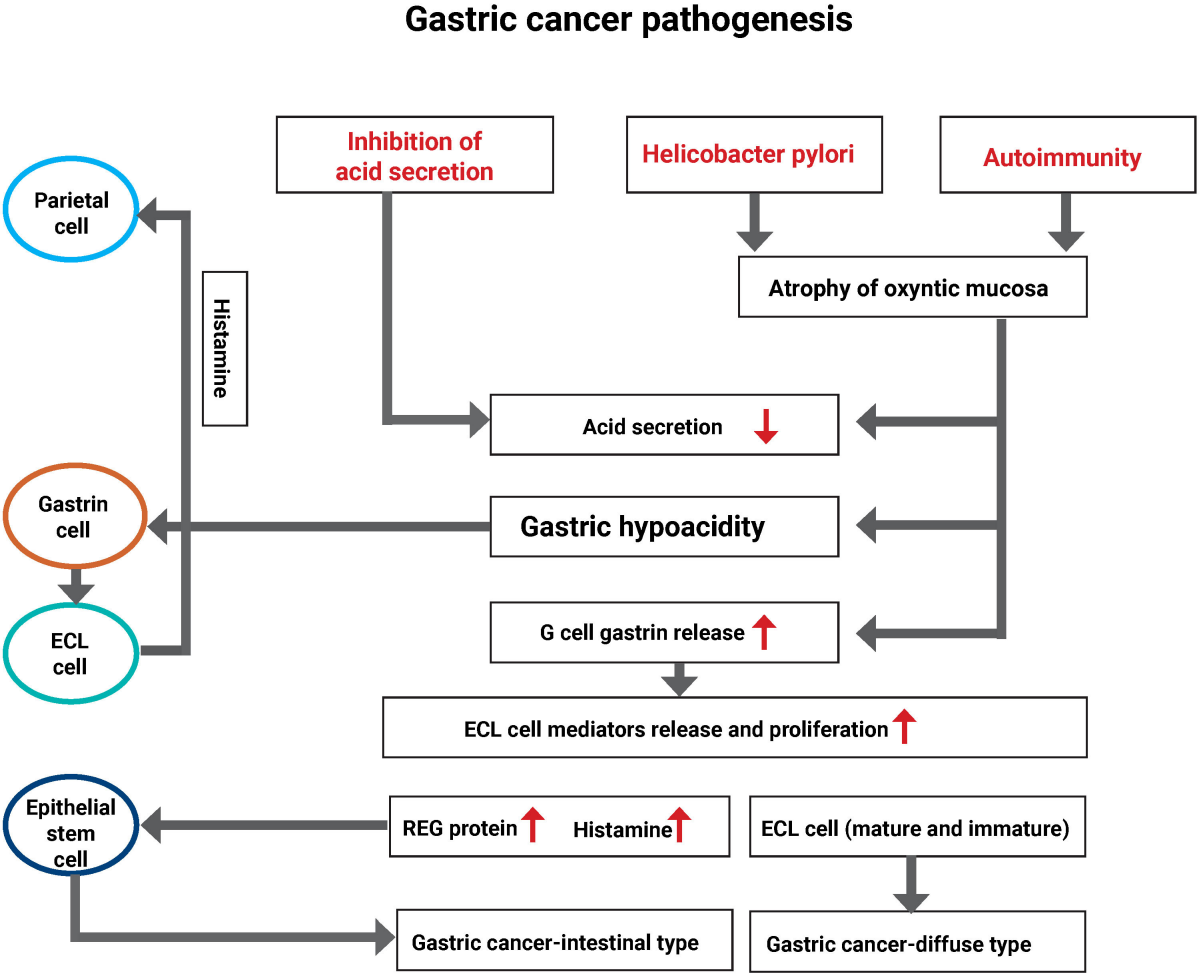
The central role of gastrin and the ECL cell in gastric carcinogenesis.

A strong body of circumstantial evidence exists to support the proposal that long-term hypergastrinemia predisposes to gastric neoplasia via its trophic effect on the ECL cell. This is summarized in **Figure 3** and **Table 1**.

**Table 1:** Gastric malignancies (gastric NETs and gastric carcinomas) secondary to gastrin.

CONDITION	PROBABILITY (strength)					
	PROVEN		PROBABLE		POSSIBLE	
	Human	Animal	Human	Animal	Human	Animal
***Profound acid inhibition***	X(69, 214, 218, 222)	X(4, 5)				
***Innate K/H ATPase defect***	X(31)					
***H. Pylori Infection***			**X**(59, 69)	**X**(70, 140)		
***Autoimmune gastritis***	X(27, 28, 44, 79, 86)					
***Gastrinoma***	X(240, 242)					
***Japanese cotton rats***		X(30, 169)				
***Mastomys natalensis***		X(163)				
***Partial gastric corpectomy***		X(132)				

**Proven:** Induced by gastrin or prevented by a gastrin antagonist/antrectomy.

**Probable:** All data indicate a central role of gastrin, but the non-availability of a gastrin antagonist precludes definitive proof .

References in parentheses.

Moreover, moderate hypergastrinemia of long duration may be carcinogenic. The stimulation of ECL cell proliferation also increases the risk of mutations as demonstrated by the development of malignant tumors by hypergastrinemia as exemplified by ECL cell gastro NETs and ECL cell gastro NECs in patients with pernicious anemia (83, 85, 212). Hypergastrinemia of short duration will also be expected to induce mutations, but with return to normogastrinemia the ECL cell including those with mutations will again show slow proliferation. However, some of the mutated cells may upon a later hypergastrinemia be stimulated to growth and even clinical neoplasia. The cancer risk of hypergastrinemia in the elderly population exposed to profound acid suppression is small since their life span is less than the long duration of exposure necessary to facilitate the development of clinical cancer. On the other hand, even mutations due to short time hypergastrinemia in children may over a lifetime be sufficient to develop into clinical cancer even with long-time normogastrinemia. It seems therefore that in children caution with exposure to profound acid inhibition should be considered.

It is plausible that H. pylori eradication in the future will involve a period of anacidity induced by a potassium-competitive acid blocker (P-CAB) and thus avoid use of antibiotics (229). A combination of P-CAB and a gastrin antagonist might accordingly be ideal in the H. pylori eradication. Furthermore, profound acid inhibition augments the hypergastrinemia due to H. pylori infection (242), and it is established that treatment with agents inducing profound acid inhibition predisposes to gastric cancer after relatively short latency after H. pylori eradication (69, 223). H. pylori should accordingly be eradicated before starting long-term treatment with a drug causing profound acid inhibition. Since each cell division is accompanied by a certain but small risk of mutation, hypergastrinemia secondary to drug induced inhibition of acid secretion will necessarily increase the number of ECL cell mutations. Therefore H. pylori eradication using drugs in doses causing near anacidity should preferably be accompanied by a gastrin antagonist.

Gastro-esophageal reflux disease is the most prevalent indication for treatment with drugs causing profound acid inhibition. In a large follow-up study of patients with gastro-esophageal reflux only those with esophagitis had an increased risk of esophageal cancer (243). This finding should have the consequence that all patients with reflux symptoms should have an upper gastrointestinal endoscopy before initiating treatment with the most efficient inhibitors of acid secretion. After all, drugs causing profound acid inhibition remove a biological function (killing of microorganisms) which has been shown to increase the risk of some bacterial diseases (244) and possibly other diseases with unknown etiology (245). Thus, use of drugs causing profound acid inhibition seems to be an unnecessary overtreatment of patients with reflux symptoms without esophagitis, and only patients with reflux esophagitis should be given profound inhibitors of acid secretion. In those with only symptoms but without esophagitis, it is reasonable to start with a histamine-2 blocker. It should be remembered that PPIs induce rebound acid hypersecretion (110) and that such treatment leads to tolerance to histamine-2 blockers via hyperplasia of the histamine producing ECL cell (246).

## Conclusion

Oxyntic atrophy due to H. pylori or autoimmune gastritis with secondary hypergastrinemia is central in gastric carcinogenesis. Long-term hypergastrinemia due to use of drugs causing profound inhibition of gastric acid secretion will naturally also lead to hypergastrinemia with a risk of tumors originating from CCK2/gastrin receptor cells, particularly the ECL cell. Taking into consideration that H. pylori infection mainly occurs in childhood and gastric cancer is a disease most often affecting middle-aged or old subjects, the observation period of PPI use to evaluate safety has been too short.

We must rely on animal studies which unequivocally have shown that hypergastrinemia is carcinogenic. Furthermore, use of drugs causing profound acid inhibition should be reduced as much as possible taking diagnosis and the age of the patient into consideration. Finally, the knowledge of the important role of gastrin in H. pylori carcinogenesis should lead to a rapid development of a gastrin antagonist for clinical us.
